# Disparities in Electronic Patient Portal Use in Prenatal Care: Retrospective Cohort Study

**DOI:** 10.2196/14445

**Published:** 2019-09-23

**Authors:** Erinma P Ukoha, Joe Feinglass, Lynn M Yee

**Affiliations:** 1 Department of Obstetrics, Gynecology, and Reproductive Sciences University of California San Francisco San Francisco, CA United States; 2 Division of General Internal Medicine and Geriatrics Department of Medicine Northwestern University Feinberg School of Medicine Chicago, IL United States; 3 Division of Maternal Fetal Medicine Department of Obstetrics and Gynecology Northwestern University Feinberg School of Medicine Chicago, IL United States

**Keywords:** patient portals, socioeconomic factors, pregnancy, cohort studies

## Abstract

**Background:**

Electronic patient portals are websites that provide individuals access to their personal health records and allow them to engage through a secure Web-based platform. These portals are becoming increasingly popular in contemporary health care systems. Patient portal use has been found to be beneficial in multiple specialties, especially in the management of chronic disease. However, disparities have been identified in portal use in which racial and ethnic minorities and individuals with lower socioeconomic status have been shown to be less likely to enroll and use patient portals than non-Hispanic white persons and individuals with higher socioeconomic status. Electronic patient portal use by childbearing women has not been well studied, and data on portal use during pregnancy are limited.

**Objective:**

This study aimed to quantify the use of an electronic patient portal during pregnancy and examine whether disparities related to patients’ demographics or clinical characteristics exist.

**Methods:**

This was a retrospective cohort study of women who received prenatal care at an academic medical center from 2014 to 2016. Clinical records were reviewed for portal use and patient data. Patients were considered enrolled in the portal if they had an account at the time of delivery, and enrollees were compared with nonenrollees. Enrollees were further categorized based on the number of secure messages sent during pregnancy as active (≥1) or inactive (0) users. Bivariable chi-square and multivariable Poisson regression models were used to calculate the incidence rate ratio of portal enrollment and, if enrolled, of active use based on patients’ characteristics.

**Results:**

Of the 3450 women eligible for inclusion, 2530 (73.33%) enrolled in the portal. Of these enrollees, 72.09% (1824/2530) were active users. There was no difference in portal enrollment by maternal race and ethnicity on multivariable models. Women with public insurance (adjusted incidence rate ratio; aIRR 0.60, 95% CI 0.49-0.84), late enrollment in prenatal care (aIRR 0.78, 95% CI 0.69-0.89 for second trimester and aIRR 0.50, 95% CI 0.39-0.64 for third trimester), and high-risk pregnancies (aIRR 0.82, 95% CI 0.75-0.89) were significantly less likely to enroll. Conversely, nulliparity (aIRR 1.10, 95% CI 1.02-1.20) and having more than 8 prescription medications at prenatal care initiation (aIRR 1.19, 95% CI 1.06-1.32) were associated with greater likelihood of enrollment. Among portal enrollees, the only factor significantly associated with active portal use (ie, secure messaging) was nulliparity (aIRR 1.11, 95% CI 1.01-1.23).

**Conclusions:**

Among an obstetric population, multiple clinical and socioeconomic factors were associated with electronic portal enrollment, but not subsequent active use. As portals become more integrated as tools to promote health, efforts should be made to ensure that already vulnerable populations are not further disadvantaged with regard to electronic-based care.

## Introduction

### Background

The use of technology to communicate between patients and providers has become routine in medicine. One such technological development is the use of Web-based patient portal services in the electronic health record, which has been adopted in large part because of the requirements of the 2009 Health Information Technology for Economic and Clinical Health (HITECH) Act. HITECH created the Electronic Health Record Incentive Programs and required meaningful use of programs such as patient portals [1]. Financial support for patient portals by the government has encouraged its widespread adoption [2].

A patient portal is a secure Web-based platform directly linked to an electronic medical record (EMR) that provides personal health information, encouraging participants to become more active in their health care [3]. Portals allow individuals to communicate with health care providers, access portions of their medical record, refill prescriptions, and schedule appointments [4]. Of the numerous studies that have explored the effect of patient portals on clinical care, many have shown portal use to be associated with positive patient-reported outcomes, including improved patient satisfaction and patient-provider communication [1,5-8]. In particular, secure messaging is a unique aspect within patient portals that may facilitate patient self-management, shared decision making, and patient satisfaction by allowing patients more opportunities to communicate with providers [9]. Secure messaging has been associated with favorable clinical outcomes, specifically in studies examining diabetes management, in which patients who engage in secure messaging have lower hemoglobin A_1c_ values [1,8,10-15].

### Objectives

The use of patient portals has not been widely investigated in obstetrics [16,17]. Moreover, with the rapid expansion of patient portals, disparities in health outcomes and care that already exist may be perpetuated by disparities in technology access and electronic health literacy [4,18-23]. Given that studies outside of obstetrics suggest portals may be associated with favorable clinical outcomes, there is an impetus to better understand the patterns of and factors associated with portal use during pregnancy. Thus, this study aimed to estimate the use of an electronic patient portal during pregnancy and determine whether disparities in portal use exist.

## Methods

### Study Cohort

This study was a cohort study of all women who received antenatal care at a single large-volume academic medical center from January 1, 2014, to January 1, 2016. Women eligible for inclusion must have been older than 18 years and received prenatal care, as defined by at least three clinical encounters, with Northwestern Medical Group providers. The study was approved by the institutional review board of Northwestern University before initiation, and all data were obtained from the EMR used for clinical care.

Northwestern Memorial Hospital and Northwestern Medical Group use EpicCare EMR and MyChart, the associated commercial patient portal. Through Northwestern’s MyChart, patients are able to view records, review laboratory and imaging results, message providers, schedule appointments, and request medication refills. A unique access code used for portal enrollment can be generated by the provider during a clinical encounter or patients can self-enroll online through their email without an access code. Patients then have to activate their MyChart account through the portal website before they are able to freely access and use portal functions. The portal can then be used via either Web-based interfaces or mobile apps. In this practice, as part of the standard clinic workflow, all patients are invited to initiate MyChart accounts at their prenatal visit through various avenues, for example, as part of their routine prenatal intake packet, in the after-visit summary from provider encounters, or through interactions with the front desk staff. Some women may already have had accounts from previous interactions within the health care system. However, beyond enrollment, there are no additional health system campaigns or initiatives designed to encourage particular aspects of portal use.

Available portal use data included whether participants had a portal account, when it was initiated, and whether they used the portal for secure messaging. Using these data, we classified participants on the basis of antenatal portal enrollment and portal use. Patients were considered to be enrolled in the patient portal if they had an account at the time of delivery, regardless of when the account was created. Patient portal enrollees were further subcategorized into active or inactive users based on their secure message use. Active users were those who had sent 1 or more secure messages during pregnancy, whereas inactive users sent 0 messages antenatally. Only patient portal use for communication with obstetric providers (physicians, nurse practitioners, certified nurse midwives, or nurses within the Department of Obstetrics and Gynecology) qualified for this analysis (ie, communication with nonprenatal care providers was not included).

### Statistical Analysis

The primary analyses focused on portal enrollment. We compared sociodemographic characteristics of portal enrollees (cases) with those of nonenrollees (controls), including age, race, ethnicity, insurance type, primary language, and neighborhood income (for which lower income neighborhood was defined as a zip code area in which the median annual household income was less than US $40,000 based on the 2015 five-year American Community Survey census data). The following clinical characteristics were also measured: parity, obesity, high-risk pregnancy (defined as a woman with any of the following characteristics: pregestational diabetes, gestational diabetes, chronic hypertension, gestational hypertension or preeclampsia, multifetal gestation, or care with a maternal-fetal medicine physician), gestational age at initiation of care, and number of prescription medications documented in the medical record at initial prenatal encounter. Comparisons were also made based on whether, once enrolled, a woman demonstrated active versus inactive use.

We used bivariable chi-square analysis and calculated unadjusted incidence rate ratios of portal enrollment and, once enrolled, of active use based on aforementioned patients’ characteristics. To calculate adjusted incidence rate ratios (aIRR), we used multivariable Poisson regression models, adjusting for potentially confounding variables with *P*<.10 in bivariable analyses and also taking into account the time in relation to when electronic portals first launched to control for potential time bias. Poisson regression models were used for the analysis as they can be a more accurate estimate for treatment effect than adjusted odds ratios when the incidence of the outcome of interest is more common [24,25]. Analyses were performed using IBM SPSS Statistics for Macintosh, Version 24.0 and Stata Statistical Software, Release 13. All analyses were 2-tailed, and *P*<.05 was used to define statistical significance.

## Results

A total of 3450 women were eligible for inclusion. Of these women, 1553 (45.01%) were non-Hispanic white (NHW), 372 (10.78%) were non-Hispanic black (NHB), 394 (11.42%) were Hispanic, 270 (7.83%) were Asian, and 861 (24.96%) were either listed as other or unknown. Overall, 3029 (87.80%) had private insurance. By zip code of residence, 437 (12.67%) of women lived in a low-income residential area, and 98 (2.84%) had a primary language other than English. In addition, 1819 (52.72%) participants were nulliparous, and 2530 (73.33%) eligible women enrolled in the patient portal (Table 1).

In bivariable analysis, compared with NHW patients, NHB and Hispanic women were significantly less likely to enroll in the patient portal. Similarly, women who were younger, had public insurance, had a low household income, or did not speak English as a primary language all had lower frequencies of portal enrollment. On multivariable models accounting for potential confounders (Table 2), women with public insurance remained significantly less likely to enroll in the patient portal compared with women with private insurance (aIRR 0.60, 95% CI 0.49-0.84). Other demographic factors did not remain associated with portal enrollment after adjusting for potential confounders.

Regarding clinical characteristics, women with high-risk pregnancies were less likely to enroll in the patient portal—56.7% (522/920) of nonenrolled versus 34.11% (863/2530) of enrolled; *P*<.001, aIRR 0.82, 95% CI 0.75-0.89. Gestational age at initiation of care was also associated with patient portal enrollment, with those who began care in the second trimester and third trimester significantly less likely to enroll. In contrast, nulliparity (aIRR 1.10, 95% CI 1.02-1.20) and having more than 8 prescription medications at the first prenatal visit (aIRR 1.19, 95% CI 1.06-1.32) were both associated with a significantly increased likelihood of enrolling (Table 2).

Of the 2530 women who enrolled in the patient portal, 1824 (72.09%) were categorized as active users (Table 3). In contrast to the differences in socioeconomic and clinical factors seen in portal enrollment, these characteristics were not associated with active portal use among enrollees (Table 4). After adjusting for potential confounders, only nulliparity remained associated with an increased likelihood of engaging in secure messaging (aIRR 1.12, 95% CI 1.02-1.23).

**Table 1:** Sociodemographic and clinical characteristics by patient portal enrollment (N=3450).

Patients’ characteristics		Patient portal, n (%)		*P* value
		Nonenrollee (n=920)	Enrollee (n=2530)	
**Age (years)**				<.001
	18-29	242 (26.3)	407 (16.09)	
	30-39	608 (66.1)	1927 (76.17)	
	≥40	70 (7.6)	196 (7.75)	
**Race/ethnicity**				<.001
	Non-Hispanic white	310 (33.7)	1243 (49.13)	
	Non-Hispanic black	169 (18.4)	203 (8.02)	
	Hispanic	147 (16.0)	247 (9.76)	
	Asian	56 (6.1)	214 (8.46)	
	Other/unknown	238 (25.9)	623 (24.62)	
**Insurance^a^**				<.001
	Private	703 (76.4)	2326 (91.94)	
	Medicaid/Medicare	188 (20.4)	105 (4.15)	
	Self-pay/uninsured	29 (3.2)	99 (3.91)	
Low household income^b^		190 (20.7)	247 (9.76)	<.001
Non-English as primary language		48 (5.2)	50 (1.98)	<.001
High-risk pregnancy^c^		522 (56.7)	863 (34.11)	<.001
Nulliparous		392 (42.6)	1427 (56.40)	<.001
Obese (body mass index ≥30 kg/m^2^)		296 (32.2)	473 (18.70)	<.001
**Gestational age at initiation of care**				<.001
	0 to 13 0/7 weeks gestational age	555 (60.3)	2188 (86.48)	
	13 1/7 to 26 6/7 weeks gestational age	231 (25.1)	273 (10.79)	
	≥27 0/7 weeks gestational age	134 (14.6)	69 (2.72)	
**Number of prescription medications at initial prenatal encounter**				<.001
	0-3 medications	539 (58.6)	1263 (49.92)	
	4-7 medications	274 (29.8)	803 (31.74)	
	≥8 medications	107 (11.6)	464 (18.34)	

^a^Insurance was determined at the patient’s first prenatal visit.

^b^Low household income was defined as patients whose zip code corresponded to a residential area in which the median household income was less than US $40,000 based on the 2015 five-year American Community Survey census data.

^c^High-risk pregnancy was defined as a patient with any of the following characteristics: type I diabetes, type II diabetes, gestational diabetes, chronic hypertension, gestational hypertension, preeclampsia, multifetal gestation, or receiving care from the Maternal-Fetal Medicine division.

**Table 2:** Unadjusted and adjusted incidence rate ratio of electronic patient portal enrollment (N=3450).

Patients’ characteristics		Incidence rate ratio (95% CI)	Adjusted incidence rate ratio (95% CI)^a^
**Age (years)**			
	18-29	Reference	Reference
	30-39	1.21 (1.09-1.35)	1.07 (0.96-1.20)
	≥40	1.17 (0.99-1.39)	1.12 (0.94-1.33)
**Race/ethnicity**			
	Non-Hispanic white	Reference	Reference
	Non-Hispanic black	0.68 (0.59-0.79)	0.88 (0.75-1.03)
	Hispanic	0.78 (9.68-0.90)	0.96 (0.83-1.10)
	Asian	0.99 (0.86-1.14)	1.05 (0.91-1.22)
	Other/unknown	0.90 (0.82-1.00)	0.99 (0.90-1.10)
**Insurance^b^**			
	Private	Reference	Reference
	Medicaid/Medicare	0.47 (0.38-0.57)	0.60 (0.49-0.84)
	Self-pay/uninsured	1.01 (0.82-1.23)	1.0 (0.81-1.22)
Low household income^c^		0.75 (0.65-0.85)	0.91 (0.79-1.05)
Non-English as primary language		0.69 (0.52-0.91)	0.80 (0.60-1.06)
High-risk pregnancy^d^		0.77 (0.71-0.84)	0.82 (0.75-0.89)
Nulliparous		1.16 (1.07-1.25)	1.10 (1.02-1.20)
Obesity (body mass index ≥30 kg/m^2^)		0.80 (0.73-0.89)	0.99 (0.89-1.10)
**Gestational age at initiation of care**			
	0 to 13 0/7 weeks gestational age	Reference	Reference
	13 1/7 to 26 6/7 weeks gestational age	0.68 (0.60-0.77)	0.78 (0.69-0.89)
	≥27 0/7 weeks gestational age	0.43 (0.34-0.54)	0.50 (0.39-0.64)
**Number of prescription medications at initial prenatal encounter**			
	0-3 medications	Reference	Reference
	4-7 medications	1.05 (0.96-1.15)	1.06 (0.97-1.16)
	≥8 medications	1.16 (1.04-1.29)	1.19 (1.06-1.32)

^a^Multivariable models were adjusted for variables with *P*<.10 in bivariable analyses: age, race/ethnicity, insurance, household income, primary language, pregnancy risk, parity, obesity, gestational age at initiation of care, number of prescription medications at initial prenatal encounter, and time.

^b^Insurance was determined at the patient’s first prenatal visit.

^c^Low household income was defined as patients whose zip code corresponded to a residential area in which the median household income was less than US $40,000 based on the 2015 five-year American Community Survey census data.

^d^High-risk pregnancy was defined as a patient with any of the following characteristics: type I diabetes, type II diabetes, gestational diabetes, chronic hypertension, gestational hypertension, preeclampsia, multifetal gestation, or receiving care from the Maternal-Fetal Medicine division.

**Table 3:** Sociodemographic and clinical characteristics by secure messaging use among patient portal enrollees (N=2530).

Patients’ characteristics		Inactive user: sent 0 messages (n=706), n (%)	Active user: sent ≥1 message (n=1824), n (%)	*P* value
**Age (years)**				.007
	18-29	121 (17.1)	286 (15.68)	
	30-39	513 (72.7)	1414 (77.52)	
	≥40	72 (10.2)	124 (6.80)	
**Race/ethnicity**				.04
	Non-Hispanic white	331 (46.9)	912 (50.00)	
	Non-Hispanic black	70 (9.9)	133 (7.29)	
	Hispanic	66 (9.3)	181 (9.92)	
	Asian	49 (6.9)	165 (9.05)	
	Other/unknown	190 (26.9)	433 (23.74)	
**Insurance^a^**				.02
	Private	653 (92.5)	1673 (91.72)	
	Medicaid/Medicare	36 (5.1)	69 (3.78)	
	Self-pay/uninsured	17 (2.4)	82 (4.49)	
Low household income^b^		81 (11.5)	166 (9.10)	.71
Non-English as primary language		15 (2.1)	35 (1.91)	.74
High-risk pregnancy^c^		279 (39.5)	584 (32.01)	<.001
Nulliparous		344 (48.7)	1083 (59.37)	<.001
Obesity (body mass index ≥30 kg/m^2^)		146 (20.7)	327 (17.92)	.11
**Gestational age at initiation of care**				<.001
	0 to 13 0/7 weeks gestational age	577 (81.7)	1611 (88.32)	
	13 1/7 to 26 6/7 weeks gestational age	96 (13.6)	177 (9.70)	
	≥27 0/7 weeks gestational age	33 (4.7)	36 (1.97)	
**Number of prescription medications at initial prenatal encounter**				.28
	0-3 medications	340 (48.2)	923 (50.60)	
	4-7 medications	223 (31.6)	580 (31.79)	
	≥8 medications	143 (20.2)	321 (17.59)	

^a^Insurance was determined at the patient’s first prenatal visit.

^b^Low household income was defined as patients whose zip code corresponded to a residential area in which the median household income was less than US $40,000 based on the 2015 five-year American Community Survey census data.

^c^High-risk pregnancy was defined as a patient with any of the following characteristics: type I diabetes, type II diabetes, gestational diabetes, chronic hypertension, gestational hypertension, preeclampsia, multifetal gestation, or receiving care from the Maternal-Fetal Medicine division.

**Table 4:** Unadjusted and adjusted incidence rate ratio of secure messaging use among electronic patient portal enrollees (N=2530).

Patients’ characteristics		Incidence rate ratio (95% CI)	Adjusted incidence rate ratio (95% CI)^a^
**Age (years)**			
	18-29	Reference	Reference
	30-39	1.04 (0.92-1.19)	1.06 (0.93-1.21)
	≥40	0.89 (0.72-1.10)	0.94 (0.76-1.17)
**Race/ethnicity**			
	Non-Hispanic white	Reference	Reference
	Non-Hispanic black	0.90 (0.75-1.07)	0.95 (0.78-1.14)
	Hispanic	0.99 (0.85-1.16)	1.02 (0.87-1.21)
	Asian	1.04 (0.88-1.23)	1.04 (0.88-1.23)
	Other/unknown	0.94 (0.85-1.16)	0.97 (0.87-1.10)
**Insurance^b^**			
	Private	Reference	Reference
	Medicaid/Medicare	0.90 (0.71-1.15)	0.99 (0.77-1.28)
	Self-pay/uninsured	1.14 (0.91-1.42)	1.09 (0.87-1.37)
Low household income^c^		0.94 (0.80-1.10)	0.99 (0.84-1.17)
Non-English as primary language		0.99 (0.71-1.38)	1.01 (0.72-1.41)
High-risk pregnancy^d^		0.92 (0.83-1.01)	0.97 (0.87-1.07)
Nulliparous		1.12 (1.02-1.23)	1.12 (1.02-1.23)
Obesity (body mass index ≥30 kg/m^2^)		0.96 (0.86-1.09)	1.02 (0.90-1.15)
**Gestational age at initiation of care**			
	0 to 13 0/7 weeks gestational age	Reference	Reference
	13 1/7 to 26 6/7 weeks gestational age	0.89 (0.76-1.03)	0.93 (0.79-1.09)
	≥27 0/7 weeks gestational age	0.76 (0.56-1.05)	0.80 (0.58-1.10)
**Number of prescription medications at initial prenatal encounter**			
	0-3 medications	Reference	Reference
	4-7 medications	0.99 (0.89-1.10)	1.01 (0.91-1.12)
	≥8 medications	0.94 (0.83-1.07)	0.97 (0.85-1.11)

^a^Multivariable models were adjusted for variables with *P*<.10 in bivariable analyses: age, race/ethnicity, insurance, household income, primary language, pregnancy risk, parity, obesity, gestational age at initiation of care, number of prescription medications at initial prenatal encounter, and time.

^b^Insurance was determined at the patient’s first prenatal visit.

^c^Low household income was defined as patients whose zip code corresponded to a residential area in which the median household income was less than US $40,000 based on the 2015 five-year American Community Survey census data.

^d^High-risk pregnancy was defined as a patient with any of the following characteristics: type I diabetes, type II diabetes, gestational diabetes, chronic hypertension, gestational hypertension, preeclampsia, multifetal gestation, or receiving care from the Maternal-Fetal Medicine division.

## Discussion

### Principal Findings

In this study of a large and diverse obstetric population, we found significant socioeconomic and clinical disparities in patient portal enrollment among pregnant women. Specifically, women who were publicly insured, medically higher risk, and late to initiate prenatal care were less likely to enroll in the electronic patient portal, whereas women who were nulliparous and taking more medications were more likely to enroll in the electronic patient portal. However, once enrolled, we found few differences in patient characteristics based on active use within the portal.

### Comparison of Results With Previous Studies

The disparities identified in this obstetric population are consistent with previous studies examining portal use in a nonpregnant population [4,18-23]. These findings are supported by a case-control study in Boston that demonstrated individuals who registered for the electronic portal were more likely to be NHW, less likely to have Medicare or Medicaid insurance, and were younger and healthier compared with nonenrollees [19]. Furthermore, studies have shown that nonwhite and Hispanic persons, those with the lowest incomes, and publicly insured and uninsured persons were less likely to activate and subsequently use their electronic patient portal account after registration [18,21,22]. Conversely, other studies have also suggested that race and ethnicity are independent risk markers for portal use. For example, a cohort study of more than 1700 individuals at Kaiser Permanente Georgia demonstrated that compared with NHW individuals, NHB individuals were less likely to register for the patient portal. These differences by race were not accounted for by differences in education, income, or internet access, although greater education and internet access were independently associated with portal registration [4]. In our study, once potential confounders were controlled for, we identified no statistically significant difference in portal use by race. This, however, may be because of incomplete race and ethnicity data. Such disparities may, in fact, exist in a better identified population cohort.

In addition, we noted differences in portal enrollment based on clinical characteristics. Notably, women with complicated pregnancies were significantly less likely to be enrolled in the patient portal. Previous studies have come to conflicting conclusions regarding patient clinical status and portal use [18-20]. It has been suggested that the *worried well* may use the portal more, which was consistent with our findings that women classified as having high-risk pregnancies complicated by diabetes, hypertensive disorders, multifetal gestations, or under the care of the Maternal-Fetal Medicine division were less likely to be enrolled in the patient portal. Ideally, given the potential benefits of portal use, enrollment should be targeted at more complicated patients who may benefit from enhanced provider-patient communication and additional support [6]. In contrast to other findings, patients with more prescription medications at the initiation of prenatal care were in fact more likely to enroll in the patient portal. If medications are properly reconciled in accordance with electronic health record use standards, it may be that these women with more medical problems, and thus more prescription medications, are more engaged in patient portals. However, it is possible that the number of medications may actually be proxy for integration into the medical system as opposed to health status and medical morbidity. In addition, women who initiated prenatal care at a later gestational age were significantly less likely to have patient portal accounts. Factors that are associated with late presentation to prenatal care—including nonwhite ethnic group, immigrant status, lower education level, lower socioeconomic status, nontraditional perception of the value of prenatal care, uninsured, unemployed, and poor reproductive health knowledge—also may play a role in whether patients are offered the electronic patient portal, their attitude toward the patient portal, or enrollment patterns [26].

Despite disparities in portal enrollment, we identified very few differences in secure messaging use. After adjusting for potential confounders, only nulliparity remained associated with an increased likelihood of engaging in secure messaging. This finding can be accounted for in that women with their first pregnancy may have more questions in general, independent from health risk. Thus, once the initial barrier to portal enrollment is overcome, patients are overall equally likely to engage and message their providers, a finding that has also been noted in studies of nonpregnant populations [23]. This finding regarding portal use underscores the importance of identifying and overcoming potential barriers that dissuade individuals from enrolling in the patient portal.

### Clinical Implications

Electronic patient portal interfaces require several steps before achieving access, and potential barriers exist at each level from being offered registration to active use [23]. A 2016 national survey study demonstrated that compared with their white and non-Hispanic counterparts, respectively, black and Hispanic individuals were significantly less likely to be offered electronic portal access by their health care provider. Moreover, individuals who are older, have poor health, and are poorly educated were also significantly less likely to be offered access by their provider and to engage in the patient portal [27]. Yet, an overwhelming majority of individuals considered online access to their personal health information important with no difference noted by race or economic status [27]. These findings highlight the role health care providers and their inherent bias may play in adoption of electronic patient portals, a critical step required to realize any benefit associated with patient portals.

Patients’ characteristics that may affect enrollment and use of an electronic portal include, but are not limited to, electronic/computer literacy, health literacy and numeracy, perceived benefit of portal use, patient preferences regarding provider communication, and trust in the health care system and electronic mediums [18,23]. In particular, health and computer literacy have both been shown to be associated with portal enrollment and long-term portal use [28-31]. High perceived health literacy along with internet access at home, high self-rated ability when using the internet, and high overall online ability have all been associated with increased likelihood of portal use—all factors that may vary among different patient populations [28,29]. Patients’ attitudes regarding the patient portal and electronic communications may also have a varying impact on its use depending on the specific population. For example, 2 qualitative studies with focus group interviews have shown that black and Hispanic patients may have negative attitudes toward the portal, were dubious regarding electronic communications and their potential benefit, and were fearful that the portal would diminish existing relationships with providers [30,31]. These studies emphasize the importance of identifying potential barriers that may dissuade individuals from engaging in the patient portal.

### Strengths and Limitations

Strengths of this study are that it included a large population of pregnant women and applied few exclusion criteria. However, given the retrospective nature of this study, any associations cannot be assumed to be causal, and there is potential for unmeasured confounding. For example, we did not have direct measures for health literacy, internet access, education level, income level, or self-care behaviors, all of which may play a role in portal engagement. In addition, at our institution, patients can enroll in the portal on their own accord without directly receiving an access code from their provider, and the exact steps leading to any given enrollment along with the temporality of enrollment are unknown. Therefore, because there is no standardized process for enrolling patients for portal access, the degree to which provider bias and discriminatory offering patterns may be related to the disparities seen in enrollment is unclear. We have identified subsets of patients less likely to enroll; thus, studies evaluating the logistics of enrollment and systems issues related to it can shed light on how to potentially increase portal use. Furthermore, although this sample was large and diverse, the patients were nonetheless receiving care at a large academic tertiary care setting, and therefore, findings may not be fully generalizable to other contexts.

### Research Implications

Future work must identify reasons for portal nonuse, develop indicators of successful outcomes of portal use, and implement potential systems-based or provider-based interventions to increase portal enrollment [32]. Efforts to expand portal enrollment in populations of greatest need may be key to improving health communication in these populations. In addition, given that secure messaging has previously been associated with positive patient outcomes in primary care settings, more studies focusing on this particular aspect are needed [1,8,10-15]. Qualitative content analysis of patient-provider electronic communication can provide information on how secure messaging within portal use may be related to perinatal, maternal, and neonatal outcomes.

### Conclusions

In summary, we identified socioeconomic and clinical disparities within portal enrollment and use during prenatal care. Disparities that were significant in regard to patient enrollment did not exist when examining subsequent secure messaging use. Thus, once initial barriers to portal enrollment were overcome, patient portal use was similar among most groups. As electronic patient portals become more integrated as tools to promote health, it is important to understand the patterns of use and the potential impact in pregnancy, especially as it relates to perinatal outcomes in already disadvantaged groups [32].

## Abbreviations

aIRR: adjusted incidence rate ratio
EMR: electronic medical record
HITECH: Health Information Technology for Economic and Clinical Health
NHB: non-Hispanic black
NHW: non-Hispanic white
